# R-YOLO: A Real-Time Text Detector for Natural Scenes with Arbitrary Rotation

**DOI:** 10.3390/s21030888

**Published:** 2021-01-28

**Authors:** Xiqi Wang, Shunyi Zheng, Ce Zhang, Rui Li, Li Gui

**Affiliations:** 1 School of Remote Sensing and Information Engineering, Wuhan University, Wuhan 430079, China; wangxiqi@whu.edu.cn (X.W.); lironui@whu.edu.cn (R.L.); whuguili@whu.edu.cn (L.G.); 2 Lancaster Environment Centre, Lancaster University, Lancaster LA1 4YQ, UK; c.zhang9@lancaster.ac.uk; 3 UK Centre for Ecology & Hydrology, Library Avenue, Bailrigg, Lancaster LA1 4AP, UK; 4 Collaborative Innovation Center of Geospatial Technology, Wuhan University, Wuhan 430079, China; 5 School of Electronic Information, Wuhan University, Wuhan 430079, China

**Keywords:** scene text detection, arbitrarily-oriented text, rotation anchor, convolutional neural network, YOLOv4

## Abstract

Accurate and efficient text detection in natural scenes is a fundamental yet challenging task in computer vision, especially when dealing with arbitrarily-oriented texts. Most contemporary text detection methods are designed to identify horizontal or approximately horizontal text, which cannot satisfy practical detection requirements for various real-world images such as image streams or videos. To address this lacuna, we propose a novel method called Rotational You Only Look Once (R-YOLO), a robust real-time convolutional neural network (CNN) model to detect arbitrarily-oriented texts in natural image scenes. First, a rotated anchor box with angle information is used as the text bounding box over various orientations. Second, features of various scales are extracted from the input image to determine the probability, confidence, and inclined bounding boxes of the text. Finally, Rotational Distance Intersection over Union Non-Maximum Suppression is used to eliminate redundancy and acquire detection results with the highest accuracy. Experiments on benchmark comparison are conducted upon four popular datasets, i.e., ICDAR2015, ICDAR2013, MSRA-TD500, and ICDAR2017-MLT. The results indicate that the proposed R-YOLO method significantly outperforms state-of-the-art methods in terms of detection efficiency while maintaining high accuracy; for example, the proposed R-YOLO method achieves an F-measure of 82.3% at 62.5 fps with 720 p resolution on the ICDAR2015 dataset.

## 1. Introduction

Texts in natural scenes, including road traffic signs, billboards, and shopping mall signs, etc. play a crucial role in our daily lives, providing essential information on society and our environment. As a prerequisite for text recognition, real-time text detection is essential in the process of text information extraction and natural language understanding. For applications with high real-time requirements, such as real-time text translation, text interpretation for assisting the visually impaired, intelligent driving and autonomous robot navigation, even a slight delay could have catastrophic consequences. Compared with standard text on documents or the internet, texts in natural scenes are discrepant, having varied sizes, font type, color, language, and orientation. Furthermore, they often have varying illumination intensities, complex backgrounds, and multiple photographing angles, causing challenges in text detection and recognition.

Over the past decade, many state-of-the-art methods have been developed to handle the tasks mentioned above [1,2,3,4,5,6,7,8,9,10,11,12,13], wherein horizontal or approximately horizontal text regions are detected with encouraging accuracy. However, text regions in natural scenes are often not horizontal, which limits the practical utility of those methods. In recent times, certain methods have attempted to address the arbitrarily-oriented text detection problem [14,15,16,17,18,19,20,21,22,23,24,25]. In general, these methods follow a two-stage strategy based on deep CNN. The detection process is as follows: first, region proposals are generated through a selective search method or a regional proposal network; then, inclined bounding boxes regression is used for inclined proposals. Despite achieving excellent accuracy, the computational efficiency of these methods in real-time applications is far from satisfactory.

To achieve high-precision and real-time detection of arbitrarily-oriented text in complex environments, we propose a one-stage text detector called Rotational You Only Look Once (R-YOLO), based on the YOLOv4 architecture [26]. Particularly, the RDIoU-NMS algorithm is proposed to increase the accuracy and reduce the error suppression of arbitrary-oriented text detection. Compared with the traditional IoU-NMS algorithm, the RDIoU-NMS algorithm not only considers the angle factor of the inclined bounding boxes but also considers the center point distance between the two boxes. We also design a rotating anchor box with angle information to represent text bounding boxes in different directions and improve the loss function of yolov4 to achieve the inclined bounding box regression. In our proposed method, like YOLOv4, Cross Stage Partial Darknet53 (CSPDarknet53) is used as the backbone network. In the CSPDarknet53 Network, the 1 × 1 convolution kernel is widely used to reduce the dimension of the feature map, thereby improving the calculation efficiency. Besides, the R-YOLO method adopts a one-step strategy, which uses a CNN network to directly predict the categories and locations of different targets without relying on the regional proposal. This makes R-YOLO one of the fastest inclined text detection methods. Compare with YOLOv4, a fourth-scale detection branch is added to the architecture of R-YOLO, which can effectively extract shallow features and fuse them with deep features, thereby effectively improve the detection ability of small-scale text.

There are two advantages of R-YOLO. First, as a one-stage method, R-YOLO can detect arbitrarily-oriented text in real-time. Second, our method has four-scale detection branches, which provides generalization capability for small-scale text detection. We choose four popular benchmark datasets (ICDAR2015, ICDAR2013, MSRA-TD500, and ICDAR2017-MLT) to evaluate the performance of the proposed method in terms of classification accuracy and computational efficiency. The major contribution of this paper can be summarized as follows:

1. A novel framework is developed to detect scene texts in arbitrary orientations using a one-stage strategy, where a fully convolutional network (FCN) is employed to generate inclined bounding boxes for text, thereby avoiding the redundant and time-consuming intermediate steps adopted in existing methods. An anchor box with rotation angle information is designed to replace the traditional axis alignment anchor box so that text detection can be adapted to any rotation angle. A new algorithm, RDIoU-NMS, is proposed to substitute the traditional IoU-NMS algorithm.
2. The 4th scale is added into the architecture of YOLOv4 to enhance the performance of detecting small-size natural-scene text.

The remainder of this paper is organized as follows. Section 2 provides an overview of related work. Details of the proposed method are presented in Section 3, followed in Section 4 by a description of numerical experiments conducted to test the performance of the proposed method. Section 5 presents our conclusions and maps out our future work.

All of our code is available publicly at https://github.com/wxq-888/R-YOLO.

## 2. Related Work

Scene text detection and recognition have been an active research topic in computer vision over the past few decades. Comprehensive surveys and detailed analyses have been conducted [27,28,29]. Traditional natural scene text detection methods rely heavily on handcrafted features to distinguish between text and non-text components in natural scene images, including methods employing sliding window (SW) and connected component (CC) techniques [1,2,3,4]. SW methods move a multi-scale detection window through all possible locations in an image and then use a pre-trained classifier to identify whether the detection window contains text. However, the SW process ends up creating a large number of redundant detection windows, which severely limits its efficiency. Among CC-based methods, Maximum Stable Extreme Regions (MSER) [13] and Stroke Width Transform (SWT) [4] are the most representative methods, where connected components are extracted as character candidates to be classified as text or non-text. The MSER method has achieved acceptable performance in ICDAR2013 [30] and ICDAR2015 [31] competitions. However, these traditional methods lag behind deep neural network-based methods in accuracy and adaptability, particularly when encountering challenging scenes such as those with low spatial resolution and geometric distortion.

Recently, with the rapid development of deep learning, natural scene text detection has entered a new era. A wide range of CNN-based text detection methods have been developed and become mainstream with tremendous success. From the perspective of the method used, text detection methods based on deep learning can be divided into three main categories: segmentation-based methods, hybrid methods, and bounding box regression-based methods.

Segmentation-based methods [14,15,16,17,18] strive to address the issue by segmenting the text region from the background and obtaining the boundary box of the text through additional steps. EAST [19] generated a text region map using a U-shape network [32]. It regressed the oriented rectangles or quadrilaterals based on the same feature to create the score map. TextFuseNet [33] considered the text detection task to be an instance segmentation task, where character-, word- and global-level features were extracted and embedded into a multi-path fusion architecture for text detection. The network has the advantage of high detection accuracy, but computational efficiency is low. Hybrid methods [20,21] used segmentation-based methods to predict the score map of the text; thereafter the text bounding box is acquired through regression.

Bounding box regression methods can be categorized into either two-stage methods or one-stage methods. Two-stage methods rely on region proposals. The most representative network is Faster R-CNN [34]. Many state-of-the-art methods such as R2CNN [22] and RRPN [23] are designed on the basis of faster R-CNN. In R2CNN [22], Region-of-Interest (RoI) Pooling with varied pooling sizes was performed several times on the axis-aligned region proposals generated by RPN [34] and the concatenated features were used to classify the proposal, where both axis-aligned box and inclined region box were estimated. R2CNN adopted a single detection scale design and cannot perform multi-scale detection tasks. RRPN [23] method incorporated the rotation factor into the region proposal network and extended the RoI pooling layer into the rotation RoI pooling layer to realize text detection rotation. In another study [35], a two-stage detection scheme based on Scale-based Region Proposal Network (SRPN) was proposed. In the first stage, three tasks of text and non-text classification, text scale estimation, and text boundary determination were performed. The second stage employed a detector to predict the text boundary boxes in text region proposals from the first stage. By contrast, one-stage methods estimate the candidate targets directly, without relying on the region proposal. Typical networks are YOLOv3 [36], YOLOv4 [26], and Single Shot Multibox Detector (SSD) [37]. TextBoxes++ [24] is an end-to-end fast scene text detector with a single deep neural network that is inspired by SSD [37]. TextBoxes++ adopted a “long” convolution kernel to predict the bounding box, where a better receptive field was acquired to cover the long text area. During the test stage, cascaded NMS was used to solve the time-consuming problem of traditional NMS calculations. However, the angle and distance of inclined bounding boxes are not considered in the cascaded NMS method, which is prone to error suppression in dense text areas. He et al. [25] proposed a single-shot text detector that utilized an attention mechanism to enhance the text area in the image and reduced background interference in the convolutional features. RRD [20] used a regression branch and classification branch to perform feature extraction for text detection. The regression branch extracted rotation-sensitive features by actively rotating the convolutional filters, whereas the classification branch extracted rotation-invariant features by pooling the rotation sensitive features. However, dual-branch feature extraction consumes a large amount of computational resources and has a limited contribution towards increasing accuracy, which cannot be satisfactory in real-time applications.

Compared with two-stage-based methods, a one-stage method regresses the bounding box directly from the convolutional feature maps without relying on the region proposal. Therefore, one-stage-based methods have an advantage in terms of computational efficiency, which is essential for fast detection in real-time applications. This paper presents a novel one-stage method (R-YOLO) for arbitrarily-oriented text detection using a fully convolutional network (FCN) model. The proposed method can not only perform multi-scale detection to obtain detection results but also conduct real-time detection tasks for real-world applications such as image streams or videos.

## 3. Proposed Method

**Figure 1:**
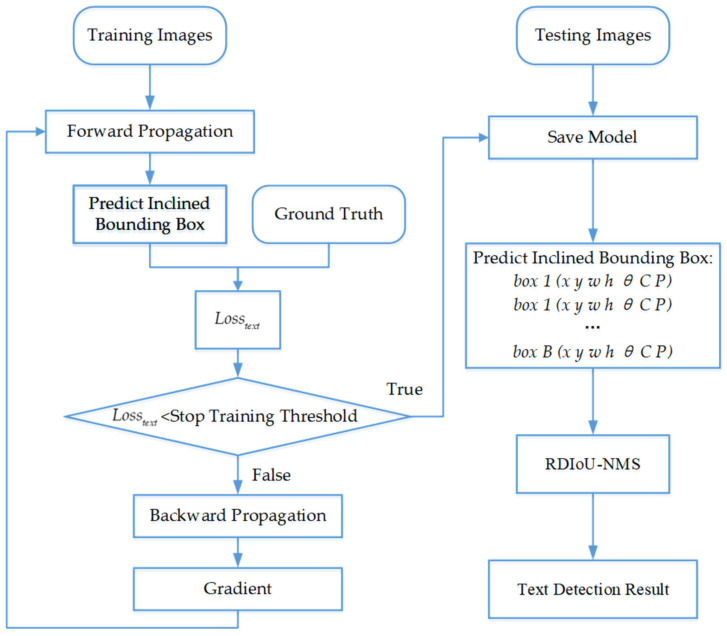
Flowchart of natural scene text detection.

In this section, the novel R-YOLO method is described in detail. R-YOLO is a detection model based on end-to-end deep learning that determines the inclined bounding boxes of the text in a natural scene image and classifies them in a single unified framework. Specifically, we have added a small-scale detection branch, proposed the RDIoU-NMS algorithm, improved the bounding box regression algorithm, and redesigned the loss function of the framework, so that it can realize text detection flexibly in natural scenes. The data processing flow is presented in Figure 1.

### 3.1. Architecture of R-YOLO

**Figure 2:**
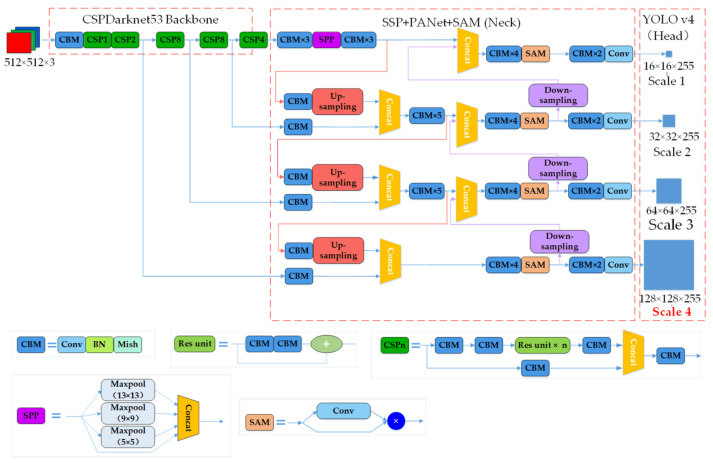
Network architecture of R-YOLO. To achieve fine-grained detection, four branches are used for object detection, with the scale of the feature map of each branch being different. Each branch predicts the confidence, class probability, and the inclined bounding boxes of the text.

YOLO is a one-stage detection model that transforms target detection into a regression problem. The YOLO family has evolved progressively from YOLOv1 to YOLOv4. Compared with YOLOv3, YOLOv4 uses several effective tricks in target detection to improve the accuracy and efficiency of target detection significantly. Figure 2 clearly describes the neural network structure of the scene text detection algorithm. In the YOLOv4 network model, CSPDarknet53 is used as the backbone. CBM is the basic component of the YOLOv4 structure, consisting of a convolutional (Conv) layer, a batch normalization (BN) layer, and a Mish activation function. A Res unit exists to construct a deeper network. Center and scale prediction (CSP) consists of three convolutional layers and *n* Res unit modules that can enhance CNN’s learning ability by dividing low-level features into two parts and then fusing cross-level features. The SPP is a spatial pyramid pooling module, which mainly transforms convolution features of different sizes into pooled features with the same length. It utilizes four scales of 1 × 1, 5 × 5, 9 × 9, and 13 × 13 for maximum pooling. As in YOLOv3, three-scale detection heads are used in YOLOv4. In our proposed method, we expand the detection branches to four to deal with the detection of small-scale scene texts.

**Figure 3:**
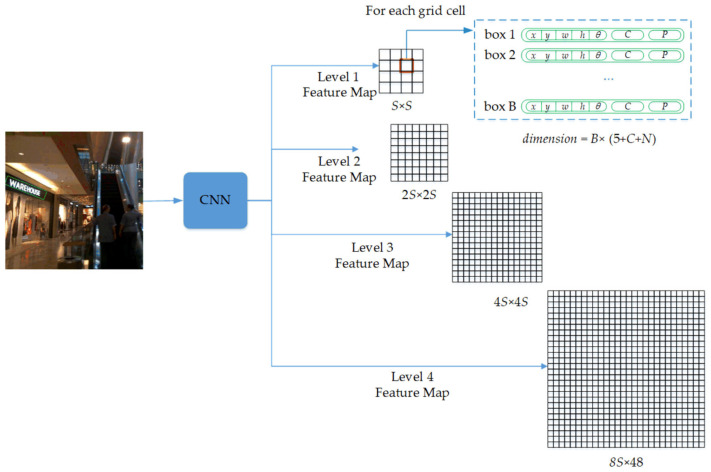
Output of R-YOLO consists of four tensors of dimension (*S*, *S*, *B* × (5 + *C* + *N*)), (2*S*, 2*S*, *B* × (5 + *C* + *N*)), (4*S*, 4*S*, *B* × (5 + *C* + *N*)), and (8S, 8S, *B* × (5 + *C* + *N*)) which correspond to the four detection levels (scales). For the ICDAR2015 dataset, *B* = 12, *C* = 1, and *N* = 1. If an input image size is 512 × 512 pixels, the outputs are four tensors of size 16 × 16 × 84, 32 × 32 × 84, 64 × 64 × 84, and 128 × 128 × 84.

R-YOLO inherits the FCN structure of YOLOv4. After the input image passes through the CNN, a feature map of four different sizes is obtained, which is divided into grids of *S* × *S*, 2*S* × 2*S*, 4*S* × 4*S*, 8*S* × 8*S* non-overlapping cells. For each cell, R-YOLO predicts the *B* inclined bounding box as illustrated by Figure 3. Each inclined bounding box contains (5 + *C* + *N*) detection attributes: five values for the position parameters (*x*, *y*, *w*, *h*, *θ*) of the inclined bounding box and *C* values for the confidence of the inclined bounding box. The confidence of text is defined as ${P\;(\mathrm{Text})\times \mathrm{RDIoU}}_{\mathrm{pred}}^{\mathrm{truth}}$. If the inclined bounding box contains text, then P (Text)=1 and the confidence will be the RDIoU between the predicted inclined bounding box and ground truth. If no text lies in the inclined bounding box, the confidence is set as 0. *N* is the number of categories in each inclined bounding box. For natural-scene text detection, *B* = 12, *C* = 1, and *N* = 1, so the output consists of four tensors of dimensions *S* × *S* × 84, 2*S* × 2*S* × 84, 4*S* × 4*S* × 84, and 8*S* × 8*S* × 84 corresponding to the four feature map levels, respectively.

### 3.2. Inclined Bounding Box Representation

**Figure 4:**
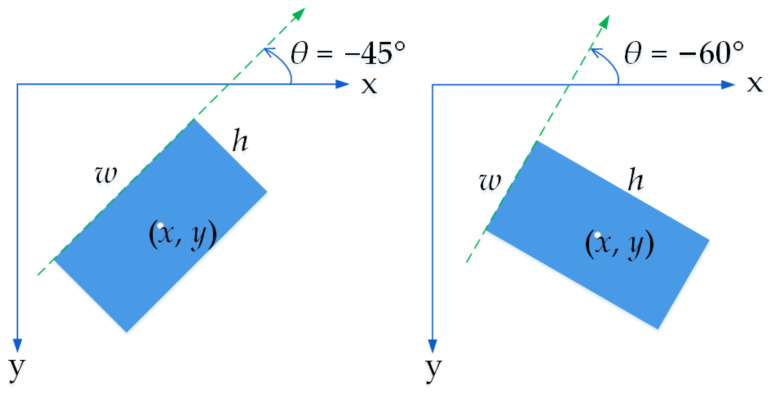
Inclined bounding box representation.

In the training stage, the ground truth of a text region set is represented by (*x*, *y*, *w*, *h*, *θ*), where the coordinates (*x*, *y*) are expressed as the coordinates of the ground-truth center point in the image coordinate system, as illustrated in Figure 4. The strategy for determining *w*, *h*, and *θ* is as follows: the edge where the *x*-axis rotates counterclockwise and first parallel to the *x*-axis is defined as *w*. The angle between *w* and the *x*-axis is *θ* and the range of *θ* is between (−90°, 0°]. The side perpendicular to *w* is denoted as *h*, so the value of *w* is not necessarily smaller than *h*. There are three advantages of using the above representation strategy. First, the uncertainty of the *θ* value due to the periodicity of the angle is eliminated. Second, it is convenient to carry out the regression operation of the inclined bounding box. Third, compared with the traditional 8-point representation of an inclined bounding box (*x*_1_, *y*_1_, *x*_2_, *y*_2_, *x*_3_, *y*_3_, *x*_4_, *y*_4_), this representation can calculate the new ground-truth value easily after rotating training images.

### 3.3. Rotation Anchor Box

**Figure 5:**
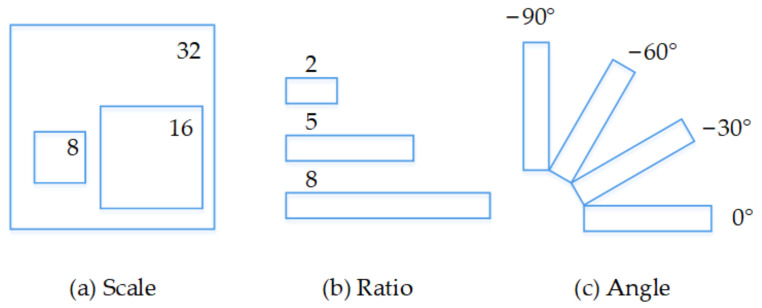
R-anchor defined in our framework.

As the ground-truth box of the text is labeled using a rectangle box with a rotation angle, the traditional anchor horizontal box, represented by scale and aspect ratio parameters only, is not suitable for text detection in natural scenes. Therefore, we design the rotation anchors (R-anchors) by adjusting several parameters. First, the scales of anchor boxes are designed to be 8, 16, and 32 pixels. Second, as the text regions usually have different scales, we define three aspect ratios of 2:1, 5:1, and 8:1 to cover the text lines with multiple aspect ratios. Furthermore, an orientation parameter is added to control the anchor direction. Four different orientations, namely, 0°, −30°, −60°, and −90° are used to ensure that the angle has the optimal initial value during the training process. The anchor strategy is summarized in Figure 5. Following the data representation steps above, an R-anchor is generated with five parameters (*x*, *y*, *w*, *h*, *θ*).

### 3.4. RDIoU-NMS

**Figure 6:**
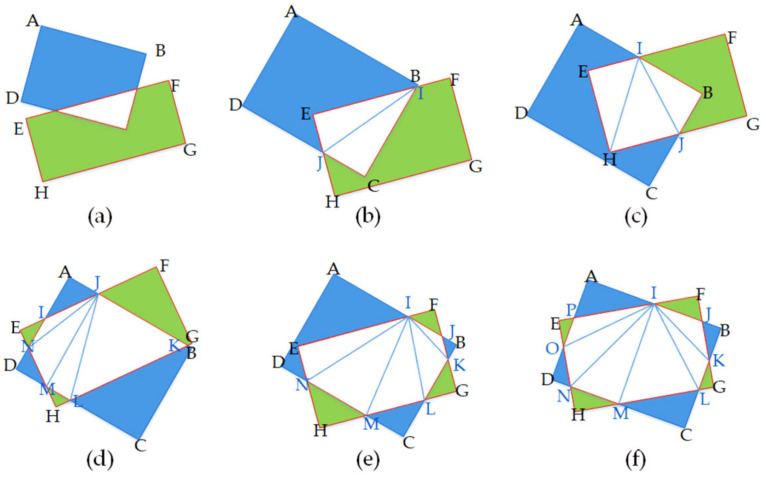
Polygon formed by the intersection of two inclined rectangles: (**a**) 3 points, (**b**) 4 points, (**c**) 5 points, (**d**) 6 points, (**e**) 7 points, (**f**) 8 points. Considering example (**c**), first, intersection points I, and J and inner vertices E, B, and H are sorted clockwise to obtain the convex polygon IBJHE, and then the intersection area *Area*(*IBJHE*) = *Area*(∆*IBJ*) + *Area*(∆*IJH*) + *Area*(∆*IHE*) is calculated.

Predicted inclined bounding boxes can be generated in any direction. The axis-aligned DIoU calculation method adopted in YOLOv4 might result in inaccuracies in the inclined bounding boxes DIoU calculation, which leads to erroneous results in the network learning process. We design a new method, named RDIoU, for the RDIoU calculation of the inclined bounding boxes, which considers not only the angle factor of the rotating bounding boxes but also the center point distance between the two boxes. The intersection of two inclined bounding boxes can create a variety of polygons, as shown in Figure 6. The vertices of the convex polygon can be sorted in the clockwise direction according to the coordinates in the image, and the triangle set can be acquired through triangulation. Taking Figure 6c as an example, the areas of all triangles are calculated and summed together. Finally, inclined *RDIoU* is derived as:

(1) $$RDIoU=\frac{Area(IBJHE)}{Area(ABCD)+Area(EFGH)-Area(IBJHE)}-\frac{\rho^{2}(b_{1},b_{2})}{c^{2}}$$

where *b*_1_ and *b*_2_ denote the central points of inclined bounding boxes *B*_1_ and *B*_2_. $\rho^{2}(b_{1},b_{2})$ is the Euclidean distance and *c* is the diagonal length of the smallest enclosing box covering the bounding box *B*_1_ and *B*_2_.

**Figure 7:**
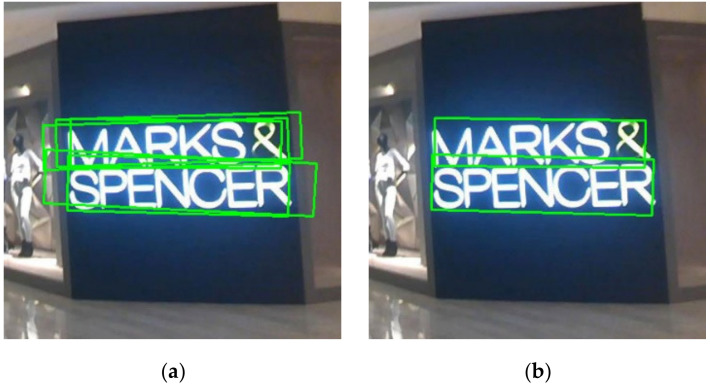
RDIoU-NMS. (**a**) All detected inclined bounding boxes; (**b**) Inclined bounding boxes after removing redundancy.

In the target detection process, a large number of inclined bounding boxes coupled with confidence are generated at the same target position and there is significant overlap between inclined bounding boxes, as illustrated in Figure 7a, where the quantification of overlap degree is expressed by RDIoU. The RDIoU-NMS algorithm is utilized to filter out redundant inclined boxes and maintain the optimal inclined boxes, as shown in Figure 7b. The list of all inclined bounding boxes is *B*, the corresponding confidence score list is C, and the overlap threshold is *N_t_*. Our goal is to get list D, which stores the optimal inclined bounding boxes. The steps of the RDIoU-NMS algorithm are as follows:

- Step 1: Sort the confidence scores in list C from large to small and adjust the order of bounding box storage in list B to make it consistent with the order of adjusted list C.
- Step 2: Take the inclined bounding box with the highest confidence as the target for comparison, delete it from list *B* and add it into the list *D* (initially *D* is empty). Calculate the RDIoU between the target inclined bounding boxes and remaining boxes in list B.
- Step 3: If the RDIoU is larger than the threshold *N*_t_, delete the bounding box from list *B*.
- Step 4: Take the inclined bounding box with the second-highest confidence as the target for comparison and repeat Steps 2 and 3 until there are no more bounding boxes left in list *B*.

The pseudocode of the algorithm is summarized in Algorithm 1.


**Algorithm 1 Calculate RDIoU-NMS**
**Input:***B* = {*b_1_*,*b_2_*, …, *b_N_*}, *C* = {*c_1_*, *c_2_*, …, *c_N_*}, *N_t_*, where *B* is the list of initial detection rotation boxes, *C* contains the corresponding detection confidence, and *N_t_* is the NMS threshold. **Output:***D*, *S*, where *D* and *S* are the list of final prediction bounding boxes and the corresponding confidence respectively. 1. **Begin** 2. *D* ← {}, *S* ← {} 3. **While**
*B* ≠ *empty*
**do** 4.  *m* ← max*C* 5.  *M* ← *b_m_*, *T* ← *c_m_* 6.  *D* ← *D* ∪ *M*, *B* ← *B* – *M* 7.  *S* ← *S* ∪ *T*, *C* ← *C* – *T* 8.  **for**
*b_i_* in *B*
**do** 9.   **if**
*RDIoU* (*M*, *b_i_*) ≥ *N_t_*
**then** 10.    *B* ← *B* – *b_i_*, *C* ← *C* – *c_i_* 11.  **end** 12. **end** 13. **return**
*D*, *S* 14. **end**

### 3.5. Learning of Text Detection

In YOLOv4, the loss function is defined as the sum of object classification loss, confidence loss, and bounding box regression loss.

(2) $$L_{\mathrm{text}}=L_{\mathrm{box}}+L_{\mathrm{confidence}}+L_{\mathrm{class}}$$

Confidence and classification loss are defined as:

(3) $$\begin{array}{l} L_{\mathrm{confidence}}=-\sum_{i=0}^{S\times S}\sum_{j=0}^{B}I_{\mathrm{ij}}^{\mathrm{obj}}\left[{\hat{C}}_{i}\log(C_{i})+(1-{\hat{C}}_{i})\log(1-C_{i})\right]\\ \;\;\;\;\;\;\;\;\;\;\;\;\;\;\;\;\;-\lambda_{noobj}\sum_{i=0}^{S\times S}\sum_{j=0}^{B}I_{ij}^{noobj}\left[{\hat{C}}_{i}\log(C_{i})+(1-{\hat{C}}_{i})\log(1-C_{i})\right] \end{array}$$

(4) $$L_{class}=-\sum_{i=0}^{S\times S}I_{i}^{obj}\sum_{c\in classes}\left[{\hat{p}}_{i}(c)\log(p_{i}(c))+(1-{\hat{p}}_{i}(c))\log(1-p_{i}(c))\right]$$

In Equation (3), *S* × *S* represents the number of cells in the feature map. *B* is the number of predictors in each grid. $I_{ij}^{obj}$ represents whether there is a target that falls in the *j*th bounding box of the *i*th grid cell. $I_{ij}^{noobj}$ indicates whether no target object falls in the *j*th bounding box of the *i*th grid cell. $\lambda_{noobj}$ refers to balancing parameters that control the trade-off between these terms. ${\hat{C}}_{i}$ and $C_{i}$ denote the true and predicted confidence, respectively. In Equation (4), $I_{i}^{obj}$ denotes if the target appears in cell *i*. ${\hat{p}}_{i}(c)$ refers to the true probability of the target, while $p_{i}(c)$ refers to the predicted value.

In the official code of YOLOv4, two types of bounding box regression loss are implemented: Mean Square Error (MSE) loss and Complete Intersection over Union (CIoU) loss. In our method, we implement inclined boundary box regression based on MSE loss. Given the angle parameter of the inclined bounding box, the calculation complexity of the angle gradient increases during the backpropagation of the CIoU loss function.

The MSE loss is defined as:

(5) $$\begin{array}{l} L_{box}=\lambda_{coord}\sum_{i=0}^{S\times S}\sum_{j=0}^{B}I_{ij}^{obj}(2-{\hat{w}}_{i}\times {\hat{h}}_{i})\left[{\left(x_{i}-{\hat{x}}_{i}\right)}^{2}+{\left(y_{i}-{\hat{y}}_{i}\right)}^{2}\right]\\ \;\;\;\;\;\;\;\;\;+\lambda_{coord}\sum_{i=0}^{S\times S}\sum_{j=0}^{B}I_{ij}^{obj}(2-{\hat{w}}_{i}\times {\hat{h}}_{i})\left[{\left(w_{i}-{\hat{w}}_{i}\right)}^{2}+{\left(h_{i}-{\hat{h}}_{i}\right)}^{2}\right] \end{array}$$

Here, $\lambda_{coord}$ is a balancing parameter with the value set to 1. $I_{ij}^{noobj}$ indicates whether no target object falls in the *j*th bounding box of the *i*th grid cell. $\left(y_{i},x_{i},w_{i},h_{i}\right)$ and $\left({\hat{y}}_{i},{\hat{x}}_{i},{\hat{w}}_{i},{\hat{h}}_{i}\right)$ represent the center coordinate, height, and width of the predicted box and the ground truth, respectively.

The CIoU loss is defined as:

(6) $$L_{box}=1-IoU+\frac{\rho^{2}(b,b^{gt})}{c^{2}}+\alpha v$$

(7) $$\alpha =\frac{v}{\left(1-IoU\right)+v}$$

(8) $$v=\frac{4}{\pi^{2}}*{\left(\arctan\frac{w^{gt}}{h^{gt}}-\arctan\frac{w}{h}\right)}^{2}$$

Here, *IoU* is the intersection over union between the predicted box and the ground truth. $\rho^{2}{(b,b}^{\mathrm{gt}})$ is the Euclidean distance, and *c* is the diagonal length of the smallest enclosing box covering the bounding boxes. $\left(w,h\right)$ and $\left(w^{gt},h^{gt}\right)$ represent the height and width of the predicted box and the ground truth, respectively.

We add an angular loss branch based on the MSE loss function to design the loss function of the inclined bounding box regression. Given a rotation anchor box *A* = (*a_x_*, *a_y_*, *a_w_*, *a_h_*, *a_θ_*) and its corresponding ground-truth box *G* = (*g_x_*, *g_y_*, *g_w_*, *g_h_*, *g_θ_*), our goal is to learn a mapping *f* such that *f*(*A*) = *P* where *P* = (*p_x_*, *p_y_*, *p_w_*, *p*_h_, *p*_θ_) is the predicted bounding box and *P ≈ G*. The definition of the mapping relationship between *A* and *P* is expressed as:

(9) $$\left\{\begin{array}{l} p_{x}=a_{w}d_{x}(A)+a_{x}\\ p_{y}=a_{h}d_{y}(A)+a_{y}\\ p_{w}=a_{w}\exp(d_{w}(A))\\ p_{h}=a_{h}\exp(d_{h}(A))\\ p_{\theta }=d_{\theta }(A)+a_{\theta } \end{array}\right.$$

**Figure 8:**
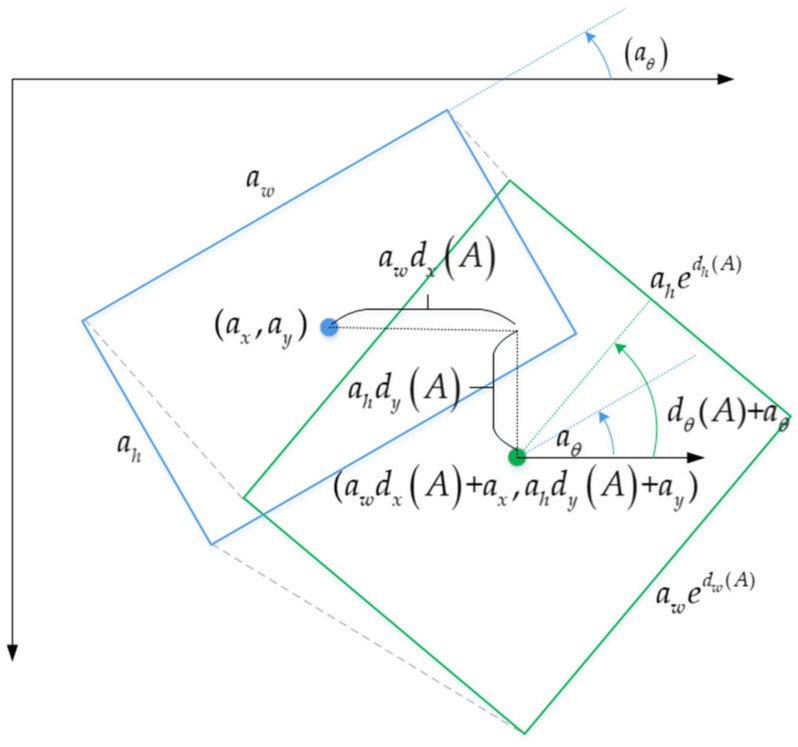
Illustration of transformation between rotation anchor box and predicted bounding box. The blue rectangle represents the rotation anchor box, and the green rectangle represents the predicted bounding box.

Here, $d_{x}(A)$ and $d_{y}(A)$ denote the scale-invariant transformation of the two centers between *A* and *P*. $d_{\theta }(A)$ represents angle-invariant transformation and $d_{w}(A)$, $d_{h}(A)$ characterize the exponential scale transformation of width and height respectively. As shown in Figure 8.

The goal of inclined bounding box regression is to train a set of parameters *W* to make *Y* = *WX*. During the training process, the input *X* is the feature map of each anchor box instead of (*a_x_*, *a_y_*, *a_w_*, *a_h_*, *a_θ_*), the feature map is represented by ϕ(A), and *Y* is calculated by the ground truth *G* and the rotation anchor region *A* to obtain the translation and zoom, expressed by $t_{*}$ where ∗ is one of (*x*, *y*, *w*, *h*, *θ*) as:

(10) $$\left\{\begin{array}{l} t_{x}=(g_{x}-a_{x})/a_{w}\\ t_{y}=(g_{y}-a_{y})/a_{h}\\ t_{w}=\log(g_{w}/a_{w})\\ t_{h}=\log(g_{h}/a_{h})\\ t_{\theta }=g_{\theta }-a_{\theta } \end{array}\right.$$

Through iterative training, *W* makes the Wϕ(A)≈t with our loss function acquired as:

(11) $$L_{box}=\lambda_{coord}\sum_{i=0}^{s\times s}\sum_{j=0}^{B}I_{ij}^{obj}\left(2-h_{i}\times w_{i}\right){\left(t_{*}^{i}-w_{*}^{T}\phi (A)\right)}^{2}$$

Here $\lambda_{coord}$ refers to balancing parameters with the value set to 1. *w*_i_ and *h*_i_ in (2 − *h*_i_ × *w*_i_) are the width and height of the ground truth, respectively. The role of (2 − *h*_i_ × *w*_i_) is responsible for balancing the generated loss value when detecting large and small objects.

## 4. Experiments

This section evaluates the proposed algorithm on standard benchmarks and compares it with several existing methods. Analysis and discussions regarding our algorithm are also presented in the details.

### 4.1. Benchmark Datasets

We selected three datasets containing directional text: ICDAR2015 [31], MSRA-TD500 [38], and ICDAR2017-MLT [39] for experiments to evaluate the performance on various directional text. To further demonstrate the versatility of R-YOLO, we also conducted experiments on a popular horizontal text dataset, ICDAR2013 [30]. A brief description of all relevant datasets is given below.

ICDAR2015 [31]: The ICDAR2015 scene text dataset issues from Challenge 4 of the ICDAR2015 Robust Reading Competition. The dataset comprises 1000 training images and 500 testing images, which were captured using Google glasses with relatively low resolutions. The text instance annotations have four vertices, which form an irregular quadrilateral bounding box with orientation information.

MSRA-TD500 [38]: The MSRA-TD500 dataset contains 200 test images and 300 training images, which contain arbitrarily-oriented text in Chinese as well as English. The texts are labeled with inclined boxes made up by the upper left corner of the rectangle, the width and height, and the rotation angle at the sentence level. Some long straight text lines appear in the dataset.

ICDAR2013 [30]: The ICDAR2013 dataset contains 233 test images and 229 training images, which is the key scene text of the ICDAR Robust Reading Competition. The scene text is horizontal and labeled with a horizontal rectangle box, including the upper left vertex and the lower right vertex of the rectangle.

ICDAR2017-MLT [39]: The ICDAR2017-MLT is a large-scale multi-lingual text dataset, which contains 7200 images for training, 1800 images for validating, and 9000 images for testing. The dataset consists of natural scene images containing texts in nine languages with multiple orientations. Some languages are labeled at line-level such as Chinese, Korean, and Japanese, while others are labeled at word-level such as English, French, Arabic, and Bangla. The different text length distributions in different languages make the detection task much more challenging.

We use five strategies to expand the training data set and to improve the robustness of training weights: (1) the image is rotated by 90, 180, and 270 degrees; (2) the image is flipped up and down, left and right; (3) the image is randomly translated jittering; (4) the brightness, contrast, hue, saturation, and noise of an image is adjusted; and (5) the mosaic data enhancement method is adopted, which randomly crops a part of four images and then puts them together into a new image.

The classical evaluation protocols for text detection, word spotting, and end-to-end recognition all rely on precision (*P*), recall (*R*), and F-measure (*F*). Precision represents the ratio of the number of correctly detected text regions to the total number of detected text regions. Recall represents the ratio of the number of correctly detected text regions to the total number of text regions in the dataset. F-measure is a single measure of quality created by combining recall and precision. These evaluation protocols are expressed as:

(12) $$\left\{\begin{array}{l} P=\frac{TP}{TP+FP}\\ R=\frac{TP}{TP+FN}\\ F=2\times \frac{P\times R}{P+R} \end{array}\right.$$

Here, *TP*, *FP*, and *FN* are the numbers of hit boxes, incorrectly identified boxes, and missed boxes, respectively.

### 4.2. Implementation Details

Our scene text detection network is initialized using a pre-trained CSPDarknet53 mode. The number of iterations depends on the size of *L_text_* (the output value of the loss function). When *L_text_* < 0.5 for a period of time, the iteration will stop. The weight decay and momentum are set to 5 × 10^−4^ and 0.9 respectively and the mini-batch size is set to 4. Testing images are resized to 512 × 512. The threshold N_t_ of RDIoU-NMS is set as 0.4. The confidence threshold and RDIoU threshold are set to 0.6 and 0.5, respectively. All the experiments are conducted on a single NVIDIA GeForce RTX 3090 graphic card with 24 GB memory, which adopts a new generation of Ampere architecture design, and its computing performance is faster than Titan X and Titan Xp.

### 4.3. Evaluation on Oriented Text Benchmark

We evaluate R-YOLO on the ICDAR2015 dataset. The model is fine-tuned for 50 k iterations on the training dataset of ICDAR2015. During the tuning stage, the learning rate starts from 1.0 × 10^−3^ and is multiplied by 1/10 after 4.0 × 10^4^ and 4.5 × 10^4^ iterations.

**Figure 9:**
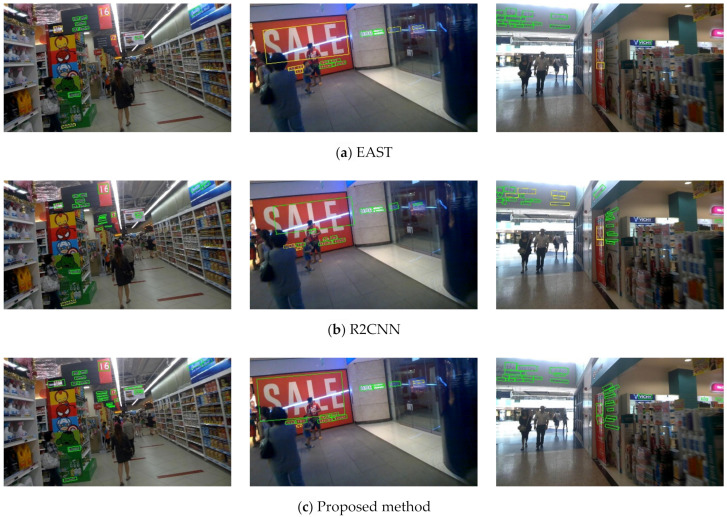
Qualitative comparisons of text detection results on some ICDAR2015 incidental text images. Green bounding boxes: correct detections; yellow bounding boxes: missed ground truths.

The quantitative results of the proposed method and other state-of-the-art methods are listed in Table 1. Our method achieves an F-measure of 82.3% and a computational speed of 62.5 fps. Compared with SegLink [18], He et al. [25], EAST [19], He et al. [40], DSRN [41], TextBoxes++ [24], and RRD [20], which are one-step methods, our F-measure is higher by 7.3%, 5.3%, 1.6%, 1.3%, 0.9%, 0.6%, and 0.1%, respectively. As regards speed, our proposed method is 3.72 times faster than the fastest method and 56.8 times faster than the slowest method listed in Table 1. This indicates that R-YOLO significantly outperforms other one-step-based methods in terms of detection efficiency and accuracy. Qualitative comparisons of text detection results are given in Figure 9.

We also beat the FTPN [43], RRPN [23], and SRPN+SRPN_Det_ [35] methods based on the two-step strategy; our method’s F-measure is higher by 9.5%, 2.3%, and 2.7%, respectively. Compared with TextFuseNet [33], which is an instance segmentation-based method, the detection accuracy of the proposed method is 9.8% lower. TextFuseNet [33] utilizes three branches to obtain three levels of features and adopts multi-path fusion architecture to obtain fused features for text detection, which consumes a large amount of computational resources, resulting in the detection speed is only 1/15 of ours. Compared with R2CNN [22] and SRPN+VGG_Det_ [35], we lose 0.2% and 3.1% accuracy. From the test results, it is obvious that there is still a gap between our approach and some two-stage-based methods in terms of detection accuracy. However, our proposed method has significant advantages in terms of detection speed. Overall, the R-YOLO method achieves comparable performance with most two-stage methods, while maintaining real-time detection speed.

### 4.4. Evaluation on Long Text Benchmark

To further test the ability of our proposed method to detect long texts, we perform fine-tuning experiments on the MSRA-TD500 dataset and stop after about 45 k iterations. During the tuning stage, training images are resized to 512 × 512. The learning rate starts from 1.0 × 10^−3^ and is multiplied by 1/10 after 3.5 × 10^4^ and 4.0 × 10^4^ iterations.

**Figure 10:**
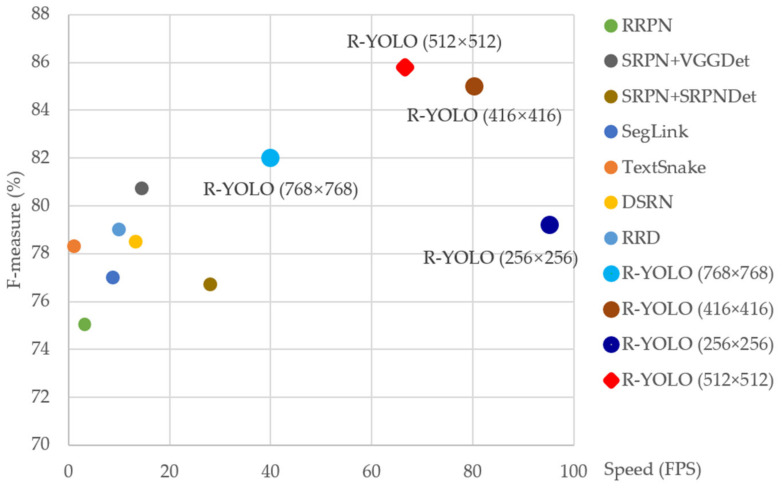
Comparisons of several recent scene text detection methods on the MSRA-TD500 dataset in terms of accuracy and speed. R-YOLO (512 × 512) (in red) achieves the ideal tradeoff between effectiveness and efficiency. Detailed results are listed in Table 2.

**Figure 11:**
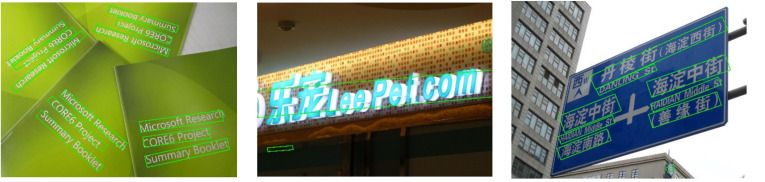

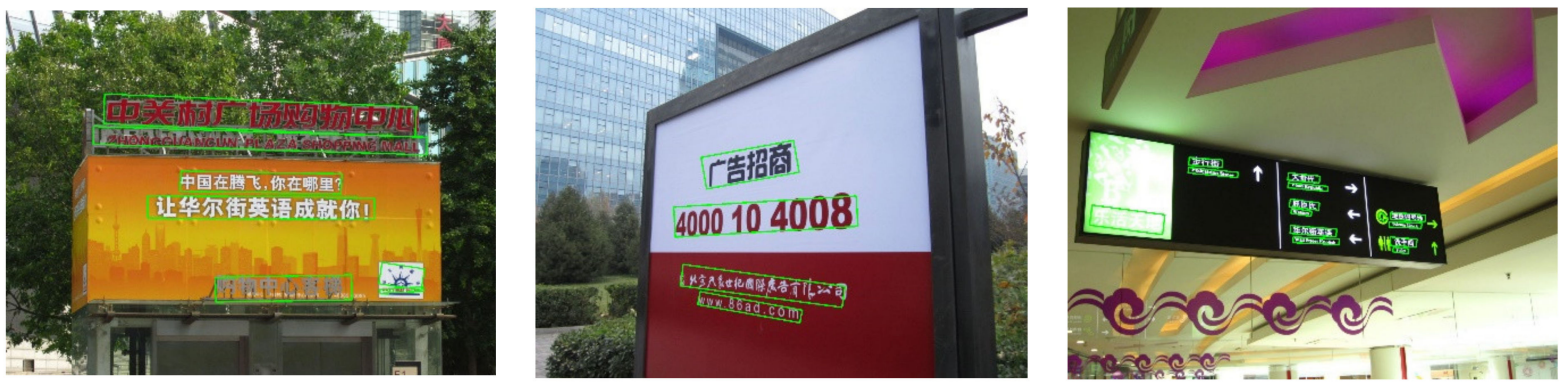
Results on MSRA-TD500.

As summarized in Table 2, testing images of four different sizes are evaluated. R-YOLO (256 × 256) achieves an F-measure of 79.2%, while the detection speed is 95.2 fps. R-YOLO (512 × 512) achieves a precision, recall, and F-measure of 90.2%, 81.9%, and 85.8% respectively, while the detection speed is 66.6 fps. From the test results, we find that the detection speed is related to the resolution of the test image. As the resolution of the test image decreases, the detection speed increases. The detection accuracy is related to the size of the training image. When the size of the test image is closer to the training image, the detection accuracy is higher. Therefore, we can resample the test image to the scale of the training image to increase the detection accuracy. Compared with SRPN+VGG_Det_ [35], which is the state-of-the-art method in terms of detection accuracy, the F-measure of R-YOLO (512 × 512) is 5.1% higher. It is also 4.5 times faster. The results show that our proposed method achieves a performance that is comparable to that of state-of-the-art methods, which means it can also process multi-oriented long texts satisfactorily. Figure 10 shows comparisons of several recent scene text detection methods. Some qualitative results are visualized in Figure 11.

### 4.5. Evaluation on Horizontal Text Benchmark

We also conducted experiments on ICDAR2013 [30] to test the general adaptability of our method. This dataset contains 233 focused scene text images where the text in the images is horizontal. During the tuning stage, the model is fine-tuned for 25 k iterations. The learning rate starts from 1.0 × 10^−3^ and is multiplied by 1/10 after 1.5 × 10^4^ and 2.0 × 10^4^ iterations.

**Figure 12:**
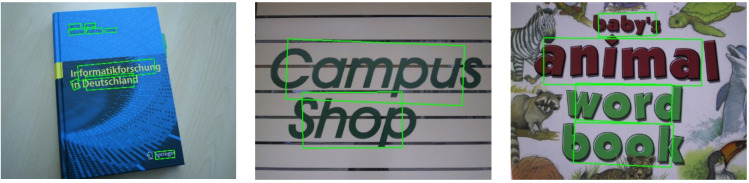

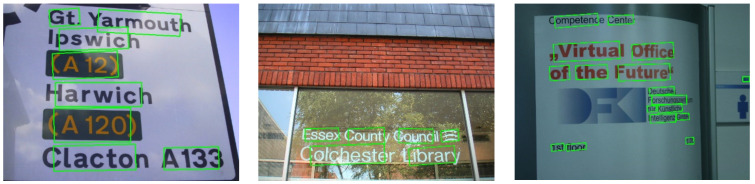
Example results of R-YOLO on ICDAR2013.

Table 3 compares the results of YOLOv4 and the proposed method. The recall rate is improved from 71.5% to 82.9%, and the F-measure is improved from 80.1% to 86.4%, while the speed reduces by 0.2 fps only. R-YOLO achieves at least 1.3% improvement over other methods except for SRPN+VGG_Det_ [35] and TextFuseNet [33] on this dataset. However, TextFuseNet [33] performs text detection by fusing three levels of features and only processes four images per second. This is not viable for real-time detection. Some detection results obtained on the benchmarks are illustrated in Figure 12, which show that our method can suitably handle horizontal text detection in natural images.

### 4.6. Evaluation on Multi-Lingual Text Benchmark

As shown in Table 4, we conduct an experiment to test the effectiveness of the fourth detection branch we added. Compared with the R-YOLO-3 method, R-YOLO-4 achieves better performance with the four-scale detection branch. The recall rate is improved from 69.5% to 71.7%, the F-measure increases from 72.9% to 74.3%, and the speed is reduced by 3.6 fps. These experimental results indicate that the fourth detection branch can effectively enhance the detection accuracy. Shallow features have higher resolution and contain more location and detailed information, which is an effective way to solve the problem of small-text detection. However, as the depth of the network increases, it is easy to lose shallow features. The fourth detection branch can effectively extract shallow features and fuse them with deep features. The fused features have rich detailed information as well as semantic information of deep features, which can effectively improve the detection ability of small-scale text.

In order to verify the effectiveness of the RDIoU-NMS algorithm, we conducted a comparative experiment between the RDIoU-NMS algorithm and the RIoU-NMS algorithm under the same conditions. Compared with the R-YOLO-RIoU method, R-YOLO-4 reduces the losses of F-measure and Recall by 2.6% and 5.4%. This demonstrates that the RDIoU-NMS algorithm can effectively reduce the missed detection rate.

**Figure 13:**
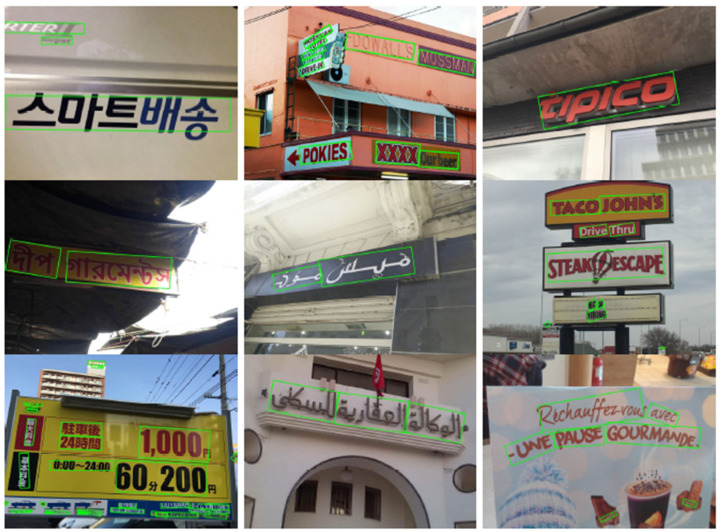
Some visualization results on the ICDAR2017-MLT benchmarks.

Compare with the previous methods, R-YOLO-4 achieves state-of-the-art results in terms of speed, surpassing the second-fastest DB-ResNet-50 [48] method at 26.6 fps. For the accuracy, R-YOLO-4 surpasses the FOTS [44], DB-ResNet-18 [48], Lyu et al. [45], LOMO [21], and CRAFT [46] methods by 3.5%, 2.6%, 1.9%, 1.2%, and 0.4%, respectively. This indicates that our method is competitive in multi-language text detection. Figure 13 demonstrates some detection results of R-YOLO-4 on ICDAR2017-MLT.

### 4.7. Analysis and Discussion

R-YOLO can achieve higher speeds than *state-of-the-art* methods because our network has two advantages. First, the proposed method adopts CSPDarknet53 as the backbone network. Compared with the existing detection methods listed in Table 1 using VGGNet or ResNet as the backbone, a large number of 1 × 1 convolution kernels are exploited in the CSPDarknet53 network to reduce the dimensions of the feature maps, which reduces the number of parameters and the size of the model considerably. Second, compared with the methods based on the two-stage strategy, our proposed method based on the one-stage strategy regresses the bounding box directly from the convolutional feature maps without relying on the region proposal, thus saving time required to calculate the region proposal.

Some qualitative comparisons are illustrated in Figure 9. From the detection results in the figure, we can observe that EAST, as well as R2CNN, missed a part of the text area and our method has achieved satisfactory detection performance. There are several reasons for this: first, EAST relies on an accurate segmentation score map as the score of the bounding boxes. However, the text region segmentation is challenging in complex environments. If the score map is not accurate enough, it is difficult to achieve accurate results. Our proposed method does not suffer from such limitations. It relies on anchor boxes and regresses the bounding boxes directly from the convolutional feature maps, where rich information is reserved as compared to the score map. Second, compared with the traditional inclined NMS algorithm used by R2CNN, a distance penalty is adopted in the proposed RDIoU-NMS algorithm to address the problem of false suppression caused by overlapping bounding boxes of different texts. In particular, in the dense text area, the effect is more satisfactory. Third, a variety of data enhancement approaches are widely adopted in our training set to improve the robustness of training weights. Fourth, in our network, effective tricks are utilized to improve the ability of network feature extraction such as SSP, PANet, and SAM, and their effectiveness has been verified in YOLOv4. The combined effect of the above-mentioned reasons makes R-YOLO more robust than competing methods in detecting arbitrarily-oriented text.

### 4.8. Limitations of the Proposed Algorithm

The proposed method outperforms the existing methods significantly in terms of detection efficiency while maintaining high accuracy, but has limitations in small-size natural scene text detection, although the addition of detection branches is helpful to improve the detection accuracy. This is a common limitation for YOLO-based object detectors. Another limitation is that the method is not good at detecting curved text.

## 5. Conclusions

In this paper, a series of improvements based on YOLOv4 are proposed to enable text detection in natural scenes where the text could be arbitrarily-oriented and of varied scales. To improve the performance of detecting small-size natural scene texts, we have added a detection branch. In order to select the optimal slanted bounding box, we proposed RDIoU-NMS, which not only considers the angle factor of the inclined bounding box but also the center-point distance between the two boxes. In addition, the representations of anchor box, bounding box regression algorithm, and loss function are improved to adapt to the detection of arbitrarily rotated text. Experimental comparisons and model analyses were conducted on the ICDAR2015, MSRA-TD500, ICDAR2013, and ICDAR2017-MLT datasets. On the ICDAR2015 dataset, our method achieved an F-measure of 82.3 at 62.5 fps with 720 p resolution. The results show that our proposed method can achieve an advanced level of text detection with very high computational efficiency. However, it still has room for improvement in terms of detection accuracy. First, the network backbone may be improved by using an advanced attention mechanism. Second, the improved loss function of inclined bounding boxes based on Pixels-IoU loss could be considered. Our future research will focus on these areas.

## Figures and Tables

**Table 1:** Results on the ICDAR2015 dataset. “*R*”, “*P*”, and “*F*” represent the recall, precision, and F-measure respectively. “OS” refers to the one-stage-based method. The FPS of the detection method running on Titan X or Titan Xp in the table is from the original paper.

Method	OS	*R* [%]	*P* [%]	*F* [%]	Device	FPS
DMPNet [42]		68.2	73.2	70.6	-	-
FTPN [43]		68.2	78.0	72.8	-	-
RRPN [23]		77.0	84.0	80.0	Titan X	4.70
R2CNN [22]		79.6	85.6	82.5	Tesla K80	2.20
SRPN+VGG_Det_ [35]		79.7	92.0	85.4	Titan Xp	16.5
SRPN+SRPN_Det_ [35]		74.8	85.2	79.6	Titan Xp	35.1
TextFuseNet [33]		89.7	94.7	92.1	Tesla V100	4.10
SegLink [18]	✓	76.8	73.1	75.0	-	-
He et al. [25]	✓	73.0	80.0	77.0	-	-
EAST [19]	✓	78.3	83.3	80.7	Titan X	16.8
He et al. [40]	✓	80.0	82.0	81.0	Titan X	1.10
DSRN [41]	✓	79.6	83.2	81.4	Titan X	8.80
TextBoxes++ [24]	✓	76.7	87.2	81.7	Titan Xp	11.6
RRD [20]	✓	79.0	85.6	82.2	Titan Xp	6.50
R-YOLO	✓	78.2	87.0	82.3	RTX 3090	62.5

**Table 2:** Quantitative results of different methods are evaluated on the MSRA-TD500 dataset. “*R*”, “*P*”, and “*F*” represent the recall, precision, and F-measure respectively. “OS” refers to the one-stage-based method. R-YOLO (512 × 512) indicates that testing images are resized to 512 × 512.

Method	OS	*R* [%]	*P* [%]	*F* [%]	FPS
RRPN [23]		69.0	82.0	75.0	3.3
SRPN+SRPN_Det_ [35]		70.8	83.6	76.7	28.1
SRPN+VGG_Det_ [35]		77.0	84.9	80.7	14.6
He et al. [40]	✓	70.0	77.0	74.0	-
EAST [19]	✓	67.4	87.3	76.1	-
SegLink [18]	✓	70.0	86.0	77.0	8.9
TextSnake [16]		73.9	83.2	78.3	1.1
DSRN [41]	✓	71.2	87.6	78.5	13.3
RRD [20]	✓	73.0	87.0	79.0	10.0
R-YOLO (768 × 768)	✓	76.5	88.3	82.0	40.0
R-YOLO (416 × 416)	✓	79.9	90.9	85.0	80.3
R-YOLO (256 × 256)	✓	71.6	88.6	79.2	95.2
R-YOLO (512 × 512)	✓	81.9	90.2	85.8	66.6

**Table 3:** Detection results on ICDAR2013. “*R*”, “*P*”, and “*F*” represent the recall, precision, and F-measure respectively. “OS” refers to the one-stage-based method.

Method	OS	*R* [%]	*P* [%]	*F* [%]	FPS
Faster R-CNN [34]		71.0	75.0	73.0	-
RRPN [23]		72.0	90.0	80.0	-
SRPN+VGG_Det_ [35]		84.2	92.5	88.2	20.9
SRPN+SRPN_Det_ [35]		83.3	86.4	84.8	30.5
TextFuseNet [33]		92.3	96.5	94.3	4.00
SSD [37]	✓	60.0	80.0	68.0	-
TextBoxes++ [24]	✓	74.0	86.0	80.0	-
YOLOv4 [26]	✓	71.5	91.0	80.1	47.2
R-YOLO	✓	82.9	90.1	86.4	47.0

**Table 4:** Detection results on ICDAR2017-MLT. “*R*”, “*P*”, and “*F*” represent the recall, precision, and F-measure respectively. OS stands for one-stage-based method. R-YOLO-3 stands for R-YOLO with three detection branches. R-YOLO-4 stands for R-YOLO with four detection branches. R-YOLO-RIoU refers to using the traditional RIoU-NMS algorithm to remove redundant inclined bounding boxes.

Method	RN	OS	*R* [%]	*P* [%]	*F* [%]	FPS
FOTS [44]			81.8	62.3	70.8	23.9
Lyu et al. [45]			74.3	70.6	72.4	-
LOMO [21]			67.2	80.2	73.1	-
CRAFT [46]			68.2	80.6	73.9	8.60
GNNets [47]			70.1	79.6	74.5	-
DB-ResNet-18 [48]			63.8	81.9	71.7	41.0
DB-ResNet-50 [48]			67.9	83.1	74.7	19.0
R-YOLO-RIoU		✓	66.3	78.0	71.7	67.5
R-YOLO-3	✓	✓	69.5	76.7	72.9	71.2
R-YOLO-4	✓	✓	71.7	77.1	74.3	67.6

## Data Availability

Not applicable.
